# Mapping of Subjective Accounts into Interpreted Clusters (MOSAIC): Topic Modelling and LLM applied to Stroboscopic Phenomenology

**DOI:** 10.1093/nc/niag008

**Published:** 2026-04-07

**Authors:** Romy Beauté, David J Schwartzman, Guillaume Dumas, Jennifer Crook, Fiona Macpherson, Adam B Barrett, Anil K Seth

**Affiliations:** School of Engineering and Informatics, Sussex Centre for Consciousness Science, University of Sussex, Falmer, Brighton, BN1 9QT, United Kingdom; School of Engineering and Informatics, Sussex AI, University of Sussex, Falmer, Brighton, BN1 9QT, United Kingdom; School of Engineering and Informatics, Sussex Centre for Consciousness Science, University of Sussex, Falmer, Brighton, BN1 9QT, United Kingdom; CHUSJ Azrieli Research Centre/Mila-Quebec AI Institute, University of Montreal, 3175 Chemin de la Côte-Sainte-Catherine, Montréal, QC, H3T 1C5, Canada; CHUSJ Azrieli Research Centre/Mila-Quebec AI Institute, University of Montreal, 6666 Rue Saint-Urbain, Montréal, QC, H2S 3H1, Canada; Canadian Institute for Advanced Research, Toronto, MaRS Centre, West Tower, 661 University Avenue, Suite 505, Toronto, ON, M5G 1M1, Canada; Collective Act, Hackney Downs Studios, 17 Amhurst Terrace, Hackney, London, E8 2BT, United Kingdom; Centre for the Study of Perceptual Experience, 67–69 Oakfield Avenue, University of Glasgow, Glasgow, G12 8QQ, United Kingdom; School of Engineering and Informatics, Sussex Centre for Consciousness Science, University of Sussex, Falmer, Brighton, BN1 9QT, United Kingdom; School of Engineering and Informatics, Sussex Centre for Consciousness Science, University of Sussex, Falmer, Brighton, BN1 9QT, United Kingdom; Canadian Institute for Advanced Research, Toronto, MaRS Centre, West Tower, 661 University Avenue, Suite 505, Toronto, ON, M5G 1M1, Canada

**Keywords:** text-based subjective reports, computational phenomenology, LLM/NLP, visual hallucinations, stroboscopic light stimulation/music, altered states of consciousness

## Abstract

Stroboscopic light stimulation (SLS) on closed eyes typically induces simple visual hallucinations, characterized by vivid, geometric, and colourful patterns. A dataset of 898 sentences, extracted from 407 open subjective reports, was recently compiled as part of the Dreamachine programme (https://dreamachine.world/) (Collective Act, 2022), an immersive multisensory experience that combines SLS and spatial sound in a collective setting. Although open reports extend the range of reportable phenomenology, their analysis presents significant challenges, particularly in systematically identifying patterns. To address this challenge, we implemented a data-driven approach leveraging large language models and topic modelling to uncover and interpret latent experiential topics directly from the Dreamachine’s text-based reports. Our analysis confirmed the presence of simple visual hallucinations typically documented in scientific studies of SLS, while also revealing experiences of altered states of consciousness and complex hallucinations. Building on these findings, our computational approach expands the systematic study of subjective experience by enabling data-driven analyses of open-ended phenomenological reports, capturing experiences not readily identified through standard questionnaires. By revealing rich and multifaceted aspects of experiences, our study broadens our understanding of stroboscopically induced phenomena while highlighting the potential of natural language processing and large language models in the field of computational phenomenology. More generally, this approach provides a practically applicable methodology for uncovering subtle hidden patterns of subjective experience across diverse research domains. Open-source implementation and an interactive web application are provided to facilitate application of this methodology.

## Introduction

Stroboscopic light stimulation (SLS) has long been recognized for its ability to transiently induce a wide range of visual hallucinations (Hewitt et al. 2025). Under closed-eyes conditions SLS reliably evokes simple (elementary) visual hallucinations, characterized by colourful geometric patterns that are devoid of semantic content (Allefeld et al. 2011, Schwartzman et al. 2019, Bartossek et al. 2021). These visual hallucinations resemble those observed in other contexts such as psychedelic states (Ter Meulen et al. 2009, Roseman et al. 2016, Schmidt and Berkemeyer 2018) and hypnagogic experiences (Bartossek et al. 2021), and pathological conditions such as migraine aura (Schott 2007, Cowan 2022), epileptic seizures (Panayiotopoulos 1994), Parkinson’s disease (Barnes and David 2001, Zarkali et al. 2019), Charles Bonnet Syndrome (Jan and Del Castillo 2012), and Psychosis (Sumich et al. 2018). Although less common, SLS has been shown to also give rise to complex visual experiences that incorporate figurative or semantic elements, such as realistic scenes, objects, and faces (Shenyan et al. 2024, Hewitt et al. 2025), potentially reflecting higher-order cortical involvement (Howard et al. 1998, Mégevand et al. 2014). Beyond visual phenomena, SLS can also alter mood, arousal, and the subjective sense of time passing (Ter Meulen et al. 2009, Bartossek et al. 2021). The ability of SLS to elicit diverse subjective experiences under controlled laboratory conditions provides a unique opportunity to investigate the neural and psychological mechanisms underlying hallucinations (Pearson et al. 2016, Rogers et al. 2021), cognitive changes (Herrmann 2001), and altered states of consciousness (ASCs) (Smythies 1959, Allefeld et al. 2011, Rule et al. 2011, Schwartzman et al. 2019). A crucial first step towards this goal is to comprehensively characterize the subjective phenomena SLS produces.

ASCs generally encompass changes in both overall conscious state and specific experiential contents, including perception, affect, cognition, and self-experience (Schwartzman et al. 2019, Bartossek et al. 2021). To our knowledge, only one study has investigated the potential of SLS to induce ASC, but did not report a significant alteration in consciousness (Bartossek et al. 2021). Specifically, a study from Bartossek (Bartossek et al. 2021) examined the effects of 3 and 10 Hz periodic SLS using well-validated questionnaires, including the ASC Rating Scale (5D-ASC/11D-ASC) (Dittrich 1998, Dittrich et al. 2010, Studerus et al. 2010) and the Phenomenology of Consciousness Inventory (Pekala 1991). Analysis of the 11D-ASC dimensions revealed predominantly elevated scores in the *Elementary Imagery* and *Complex Imagery* dimensions. The 5D-ASC results indicated the strongest effects in *Vigilance Reduction* and *Visionary Restructuralization*, with minimal effects on *Oceanic Boundlessness*. The Phenomenology of Consciousness Inventory did not reveal any notable alterations in consciousness compared with a control condition, suggesting limited overall alteration in consciousness. These findings suggest that within the laboratory, periodic fixed frequency SLS primarily induces specific visual phenomena (e.g. simple patterns and colours) and changes in vigilance, rather than broader alterations in consciousness (Schmidt and Berkemeyer 2018, Bartossek et al., 2021).

However, traditional ASC questionnaires rely on predefined dimensions, which may not fully capture unexpected or idiosyncratic experiences, potentially overlooking less common or individually distinctive aspects of ASC. To address these limitations and capture the full spectrum of stroboscopically induced phenomenology without assumptions about the types of experience that may be present, we adopted a data-driven approach. We analysed 898 sentences extracted from 407 free-form open reflections of SLS experiences collected as part of the Dreamachine programme (https://dreamachine.world/). The Dreamachine is an immersive multisensory experience that uses stroboscopic lighting and 360-deg spatial sound in a highly curated context. It ran in the four capital cities of the UK in 2022, reaching tens of thousands of people, who underwent the experience in collective settings (groups of 20–30 people).

Central to our approach is topic modelling (TM), a statistical method for identifying abstract themes in text data (Blei et al. 2003, Blei 2009). TM detects patterns of co-occurring words, grouping them into topics that reveal latent semantic structures. This technique offers a more granular exploration of text-based subjective data, capturing elements that traditional structured questionnaire assessments may overlook. By removing the constraints of predefined categories, TM allowed us to explore a wider spectrum of stroboscopically induced phenomenology within the Dreamachine dataset. We hypothesized that this approach would uncover experiential dimensions not previously identified through structured assessment, revealing latent experiential categories within the Dreamachine dataset.

We also make a methodological contribution by implementing and documenting an open-source natural language processing (NLP)-based pipeline specifically implemented for the analysis of phenomenological free text reports (MOSAIC: Mapping Of Subjective Accounts into Interpreted Clusters) (https://github.com/romybeaute/MOSAIC). Our approach bridges qualitative and quantitative research, providing a systematic, scalable method for analysing rich subjective textual data. To encourage widespread adoption, we provide a fully documented implementation of our analytical workflow, from preprocessing to interpretation. While we apply this approach to stroboscopically induced phenomenology, MOSAIC has broader applications across fields that require systematic analysis of complex and challenging-to-interpret free text reports. We outline the necessary adaptations for different datasets and discuss potential limitations. Alongside this pipeline, we provide an interactive web application (https://huggingface.co/spaces/romybeaute/MOSAICapp) enabling researchers to apply MOSAIC to their own phenomenological datasets without requiring programming expertise.

## Materials and methods

### Data description

The dataset comprises free text reports collected from participants attending the Dreamachine. The Dreamachine is an installation designed to ‘deliver an immersive and multisensory experience that uses flickering white light and 360-degree spatial sound to create a kaleidoscopic and technicolour world behind closed eyes’ (https://dreamachine.world/experience/). Visitors attended sessions in a purpose-designed venue intended to foster a calm and reflective atmosphere. The space accommodated up to 30 participants per session, with each person lying reclined on a bench arranged in a circular layout (see Figure S1).

To accommodate participants with sensitivity to flashing lights (e.g. photosensitive epilepsy, sensory sensitivity), two versions of the Dreamachine experience were developed. The ‘High Sensory’ version consisted of an association of stroboscopic lighting and a 360-deg spatial sound experience. Prior to entering the experience, all participants received a standard safety briefing regarding stroboscopic light (e.g. warnings for photosensitive epilepsy) and the nature of the immersive installation. While participants were informed about the potential for visual phenomena, they were not explicitly primed with specific imagery descriptions or expected outcomes. The total experience lasted $\sim $30 min, which included time for a short tester session to make sure participants were comfortable with the strobe lighting, and a short guided meditation to ease the participant into the experience. The SLS sequence itself lasted 18 min and was delivered by commercial-grade stroboscopes and comprised stimulation frequencies ranging from 6.9 to 15.1 Hz, with duty cycles between 37% and 100% (42–173 ms), and luminance levels up to 2500 lux. In this version, participants were instructed to keep their eyes closed throughout the duration of the experience.

The ‘Deep Listening’ version offered a parallel experience without stroboscopic stimulation, instead using sequences of coloured (non-stroboscopic) wash lights. It employed the same musical soundtrack as the High Sensory version, creating an immersive audio environment that encouraged relaxation and inward focus. In this version, participants were invited to keep their eyes either open or closed for the duration of the experience.

Following their Dreamachine experience, participants were invited to optionally complete the digital survey using a custom tablet application. This survey, designed in collaboration with Collective Act to capture a wide range of experiences, consisted of two main components: first structured questionnaires for specific categories (with some category-specific freeform response options) and then a space for general open-ended reflection. Our study focused specifically on the free-text from the general open-ended reflections, where participants provided detailed descriptions of their experiences without any specific constraint. The open-ended reflection interface presented participants with a blank space on a tablet, prompted by the following text: ‘In Dreamachine I experienced$\ldots $’.

The study received ethics approval from the University of Sussex (ethics application ID: ER/ANIL/5).

### Methods

To identify the most prevalent ‘dimensions’ of experiences present in the data, we employed TM, conducting separate analyses on the High Sensory and Deep Listening datasets. Analyses were conducted separately to allow the unique phenomenological structure of each condition to emerge independently, avoiding biases that could arise from combining datasets of different sizes and thematic content. TM is an NLP technique that identifies patterns within text by grouping words and phrases into clusters, or ‘topics’, revealing hidden or underlying themes across datasets. These *experiential topics* (we define ‘experiential topics’ as thematic clusters identified through our TM analysis that represent distinct patterns in participants’ phenomenology) represent recurring themes or patterns in participants’ subjective reports, providing a structured method of categorizing and understanding the diverse range of experiences.

TM can be implemented using various methods, including traditional techniques such as latent Dirichlet allocation (LDA) (Blei et al. 2003) and more recently, advanced models based on transformer architectures (Thompson and Mimno 2020, Vaswani et al. 2017). LDA groups topics by identifying word co-occurrences, but lacks context-awareness, defined as the ability to interpret how a word’s meaning changes based on the context of surrounding words and its position in a sentence, thus limiting its ability to capture nuanced relationships between words within sentences.

To overcome LDA’s context limitations, we used an approach combining BERTopic (Grootendorst 2022) with large language model (LLM)-based topic labelling. BERTopic addresses these limitations through its transformer-based architecture, enabling context-aware analysis of text data and capturing subtle experiential dimensions by incorporating a contextual understanding of word meanings based on their usage in sentences. For instance, although LDA might treat e.g. ‘*time*’ as the same way in different contexts (e.g. ‘*it was different this time*’ vs. ‘*I felt a distortion of time*’), BERTopic’s embeddings differentiate generic references for phenomenological implications, thus capturing the multifaceted nature of subjective experiences more accurately. Finally, we integrated Meta’s Llama-3-8B-Instruct LLM (Grattafiori et al., 2024), to provide automatic, data-driven topic interpretation and labelling based on BERTopic’s extracted keywords and sentences, rather than relying on subjective manual label generation by the researcher.

#### Data preprocessing

While BERTopic can handle raw text with minimal preprocessing, our analysis nonetheless required specific preprocessing steps to optimize topic identification. Our preprocessing involved two main stages: LLM-based text normalization followed by sentence segmentation and filtering. First, to address common issues in free-text data such as spelling errors and formatting artefacts, we employed an LLM for text cleaning, using a local instance of Meta’s Llama-3-8B-Instruct model. To preserve the privacy and confidentiality of the participants’ experiential reports, the model was run entirely on a local machine, ensuring no data were transmitted to external third-party servers. The model was prompted to act as an expert data cleaner, with strict instructions to correct spelling and remove artefacts while explicitly being told not to alter the original meaning or punctuation of the text. The quality of this automated normalization step was then verified through careful manual inspection of a random sample, which confirmed the model successfully corrected errors without distorting or modifying the participants’ reports.

Second, given the multifaceted nature of experience reports, we implemented sentence-level segmentation to enable the differentiation of distinct phenomenological themes within individual reports. This was achieved using the PunktSentenceTokenizer from the NLTK library (Bird et al. 2009). This methodological choice enables a more granular analysis of experiential reports, allowing each sentence to serve as a distinct unit for embedding and subsequent TM, enhancing the model’s ability to capture contextual nuances and semantic meaning (Reimers and Gurevych 2019). After sentence-based segmentation, we further refined the dataset by removing sentences with fewer than two words and any potential duplicate sentences to reduce noise and ensure data quality.

While we acknowledge that some individual sentences may still contain multiple experiential facets, we chose the sentence as the optimal unit of analysis to balance the need for thematic granularity with the preservation of essential semantic context, which would be lost with smaller textual units.

The final count of free text reports and resulting preprocessed sentences are displayed in Table 1. After preprocessing, the High Sensory datasets contained 700 sentences, segmented from 315 reports. The Deep Listening dataset resulted in 198 sentences, segmented from 92 reports.

**Table 1:** Summary of the number of reports per participant and number of resulting sentences (after preprocessing)

**Dataset**	**Number of reports**	**Number of sentences**
DL	92	198
HS	315	700

To characterize the richness of the dataset, descriptive statistics for the reports are provided in Table 2.

**Table 2:** Descriptive statistics for the original, non-preprocessed free-text reports, mean, standard deviation (SD), minimum (Min), and maximum (Max) values are shown for word and sentence counts for both the High Sensory (HS) and Deep Listening (DL) datasets

**Dataset**	**Metric**	**Mean**	**SD**	**Min**	**Max**
HS ($n=333$)	Word count	25.36	26.31	1	153
	Sentence count	2.21	1.77	1	10
DL ($n=98$)	Word count	24.76	26.49	1	161
	Sentence count	2.12	2.04	1	15

#### BERTopic

**Fig. 1.**
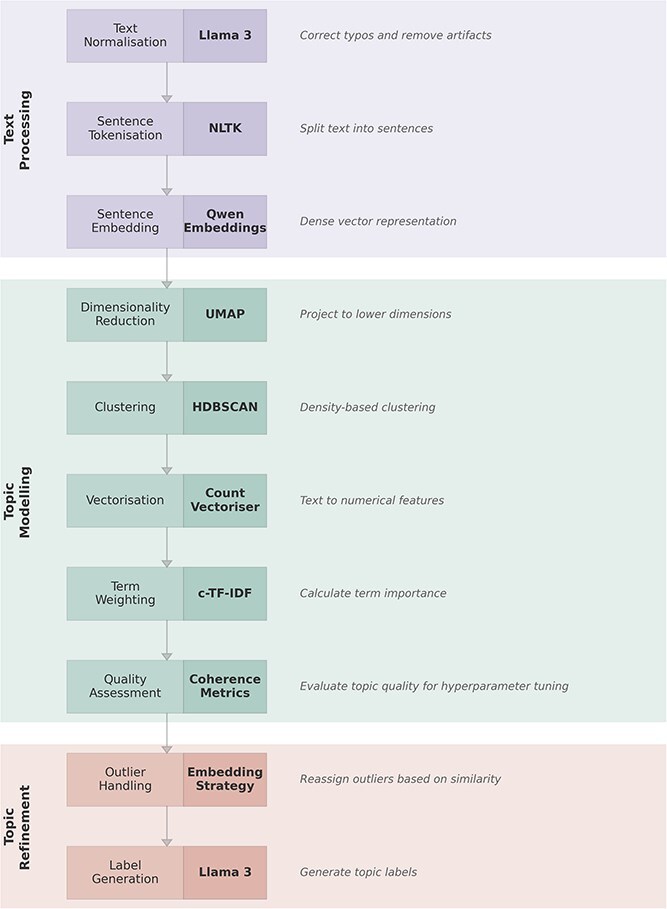
MOSAIC TM pipeline architecture comprising three phases: text processing (Llama 3 for text normalization, NLTK for sentence segmentation, and Qwen for dense document embeddings), topic modelling (UMAP for dimensionality reduction, HDBSCAN for clustering, Count Vectoriser and c-TFIDF for term weighting, and coherence metrics for quality assessment), and topic refinement (reassigning outliers using embedding similarity and generating topic labels with Llama 3).

Our analysis pipeline is built on BERTopic, a multi-stage process that begins by generating sentence-level text embeddings (Grootendorst 2022). The complete architecture of the MOSAIC pipeline is illustrated in Fig. 1. The architecture of BERTopic comprises four key components: (i) a transformer embedding model; (ii) Uniform Manifold Approximation and Projection (UMAP) dimensionality reduction (McInnes et al. 2018); (iii) Hierarchical Density-Based Spatial Clustering of Applications with Noise (HDBSCAN) (McInnes et al. 2017) clustering; (iv) cluster tagging using class-based Term Frequency-Inverse Document Frequency (c-TF-IDF). This combination produces dense clusters that are easily interpretable and coherent, while retaining important words in the topic descriptions. We detail each of these core components in the following section.

##### Transformer embeddings

The first step of our analysis involved sentence embedding (Reimers and Gurevych 2019), where each sentence from the open reports was converted into a high-dimensional vector representing its semantic context. For this, we selected the ‘Qwen/Qwen3-Embedding-0.6B’ Sentence Transformer model from the sentence-transformers library of the Hugging Face Hub (https://huggingface.co/Qwen/Qwen3-Embedding-0.6B), which was chosen for its excellent performance on the Massive Text Embedding Benchmark (MTEB) leaderboard (https://huggingface.co/spaces/mteb/leaderboard) (Muennighoff et al. 2022) for both clustering and semantic similarity tasks. This process resulted in a 1024D vector for each sentence, providing a rich semantic representation essential for the subsequent analysis of phenomenological nuances.

##### Dimensionality reduction, HDBSCAN clustering, and hyperparameter optimization

After obtaining the high-dimensional embeddings, we used a two-step process to identify topic clusters. First, dimensionality reduction was performed using UMAP(implemented using python library https://pypi.org/project/umap-learn/) (McInnes et al. 2018). UMAP was chosen for its ability to preserve both local and global data structure, which is crucial to maintaining semantic relationships in the reduced dimensional space. Following this, we employed HDBSCAN for cluster analysis (McInnes et al. 2017). A key advantage of HDBSCAN is that it does not require the number of clusters to be prespecified, instead, this number is an emergent property of the data’s density structure, which is influenced by the hyperparameters we tune.

To find the optimal combination of hyperparameters for these algorithms, we implemented a Bayesian optimization process using the Optuna framework (https://github.com/optuna/optuna), which is more efficient and robust than a traditional grid search. Rather than exhaustively testing all predefined parameter combinations, Bayesian optimization builds a probabilistic model to predict which hyperparameters are most likely to improve a set of coherence scores. This process allowed us to explore a wider and more continuous range of parameter values to find a more optimal solution in fewer trials. Separate optimization studies were conducted for the High Sensory and Deep Listening datasets, systematically tuning key UMAP and HDBSCAN parameters. For UMAP, the following parameters were tuned: n_components, controlling the dimensionality of the reduced space, n_neighbors, balancing local and global structure preservation, and min_dist, determining the minimum distance between points in the embedded space. For HDBSCAN, we tuned min_cluster_size and min_samples, controlling the minimum cluster size granularity and noise tolerance, respectively.

The primary objective for the optimization was to maximize simultaneously two evaluation metrics: a sentence embedding-based coherence score ($C_{\mathrm{embed}}$) and a traditional word-based ($C_{\mathrm{v}}$) coherence score.

Embedding coherence ($C_{\mathrm{embed}}$): We defined this metric as the average intra-topic cosine similarity of sentence embeddings within each identified cluster, which directly assesses the semantic coherence, or compactness of the identified topic clusters in the embedding space. For a given topic $T_{k}$ containing $N_{k}$ sentence embeddings, we first calculate its intra-topic coherence ($C_{\mathrm{intra}}$). This score represents the average cosine similarity between all unique pairs of sentence embeddings $(\mathbf{e}_{i}, \mathbf{e}_{j})$ within that single topic $T_{k}$: 


(1) \begin{align*}& C_{\mathrm{intra}}(T_{k}) = \frac{1}{\binom{N_{k}}{2}} \sum_{1 \le i < j \le N_{k}} \frac{\mathbf{e}_{i} \cdot \mathbf{e}_{j}}{||\mathbf{e}_{i}|| \cdot ||\mathbf{e}_{j}||}\end{align*}

The final, overall embedding coherence score ($C_{\mathrm{embed}}$) is then calculated as the mean of these individual $C_{\mathrm{intra}}$ scores across all $K$ topics found by the model (excluding outliers): 


(2) \begin{align*}& C_{\mathrm{embed}} = \frac{1}{K} \sum_{k=1}^{K} C_{\mathrm{intra}}(T_{k})\end{align*}

A high score means the sentences in the topic are all very similar in meaning. This metric was chosen as it aligns directly with the transformer-based approach, evaluating topic quality based on semantic proximity within the sentence embedding space, rather than only word co-occurrence, as it is the case for $C_{v}$ (see below).

Topic coherence ($C_{v}$): The second objective was to maximize the $C_{v}$ score (Mimno et al. 2011, Lau et al. 2014, Röder et al. 2015, Newman et al. 2010), a widely used word-based metric of topic quality. This metric quantifies the meaningfulness of relationships among the top words of each topic, and is particularly valued for showing strong correlation with human judgements of topic quality and interpretability (Röder et al. 2015). We computed $C_{v}$ using Gensim’s CoherenceModel ($C_{v}$) (https://pypi.org/project/gensim/), which (i) constructs a sliding-window word co-occurrence graph, (ii) computes normalized pointwise mutual information for word pairs among the top-$N$ words for each topic, and (iii) aggregates (by averaging) the pairwise cosine similarity scores calculated between the context vectors for each of the topic’s top words (Röder et al. 2015).

By optimizing for both objectives, we aimed to find a balanced solution, identifying topics that are both semantically compact in the embedding space ($C_{embed}$) and lexically interpretable based on word co-occurrence statistics ($C_{v}$). A multi-objective search was chosen, which systematically explores the trade-off between these two metrics by optimizing them jointly in a Pareto sense (i.e. searching for configurations that are not overly dominated by one single metric). We performed this two-objective Bayesian optimization with Optuna search was guided by the NSGA-II (Non-dominated Sorting Genetic Algorithm II) sampler (https://optuna.readthedocs.io/en/stable/reference/samplers/generated/optuna.samplers.NSGAIISampler.html) over 100 trials for both Deep Listening and High Sensory datasets, ensuring a robust search for the optimal parameter set. This number of trials was chosen as it allowed for a thorough exploration of the parameter space, with objective scores consistently reaching a stable plateau well before the final trial (Figure S2). This multi-objective process identified a set of optimal trade-off solutions (the Pareto front). To select a single, final hyperparameter configuration from this set, we then prioritized $C_{embed}$ over $C_{v}$ as it directly evaluates the semantic proximity of the embeddings themselves. The configuration reported in Table 3 is this selected solution, which yielded the highest embedding coherence ($C_{embed}$) score from this trade-off set. For the High Sensory version, the selected model achieved a $C_{embed}$ score of 0.61 and a $C_{v}$ score of 0.45, resulting in 12 topics. For the Deep Listening version, the chosen model achieved scores of 0.56 ($C_{embed}$) and 0.51 ($C_{v}$), resulting in seven topics. These coherence scores fall within the acceptable range typically observed in TM studies (0.4–0.7) (O’Callaghan et al. 2015, Röder et al. 2015).

**Table 3:** Optimal hyperparameters identified by the Optuna optimization study for the two versions of the Dreamachine: High Sensory (HS) and Deep Listening (DL)

	n_comp	n_neigh	min_dist	min_clust	min_samp	Cembed	Cv	n_topics
HS	20	26	0.015	10	8	0.61	0.45	12
DL	7	8	0.04	10	9	0.56	0.51	7

##### LLM-based topic labelling

To enhance topic interpretability after cluster identification by BERTopic, we used an LLM (Llama-3-8B-Instruct) (Grattafiori et al., 2024), to automatically generate descriptive labels for each emergent topic. To do this, we implemented a custom labelling strategy to improve label quality and context-awareness. This involved creating a comprehensive input for the LLM by supplementing the default c-TF-IDF keywords with additional sets generated via KeyBERTInspired (https://pypi.org/project/keybert/) and Maximal Marginal Relevance (MMR) (https://maartengr.github.io/KeyBERT/api/mmr.html). This collection of keywords, along with representative sentences from each topic, was then passed to the Llama-3 model. A custom prompt directed the model to synthesize these inputs into a single, precise label. This approach resulted in labels that are more nuanced and semantically grounded in the specific content of the participants’ reports.

This overall approach was intended to shift the labelling process from a subjective manual task to a data-driven method implemented by the LLM. Since LLMs are generative models, some inherent stochasticity exists, meaning that repeated labelling attempts multiple times may produce slight variations in phrasing (e.g. ‘Imagery’ vs. ‘Visuals’). However, because the same enriched inputs were used each time to generate the labels, the core semantic theme remained stable across runs. To verify label reliability, we conducted multiple labelling attempts and confirmed that variations were limited to wording rather than underlying conceptual meaning.

##### Model stability and reproducibility

To assess the structural stability of the identified topics, we conducted a bootstrapping analysis. We performed 100 iterations; in each iteration, the topic model was trained on a random subsample of 80% of the sentence corpus without replacement. We then aligned the resulting topics with the original model’s topics by calculating the cosine similarity between their centroids. This analysis assesses topic-level semantic stability, rather than sentence-level reassignment, which is expected to vary under subsampling. High cosine similarity between a bootstrapped centroid and an original centroid indicates that the semantic ‘centre’ of the cluster occupies a similar region in the embedding space; in other words, that a semantically equivalent topic emerges from the partial data. The analysis revealed a high degree of stability, with a mean cosine similarity of 0.94 ($SD = 0.05$) for the High Sensory condition and 0.93 ($SD = 0.03$) for the Deep Listening condition. These results confirm that the same semantic topics consistently re-emerge when substantial portions of the data are removed, indicating that the topic structure is driven by robust patterns in the data rather than stochastic effects. Per-topic stability scores are reported in Supplementary Table S2.

All data analysis, TM, and visualizations were implemented using Python (v3.12.3). Data handling and manipulation were performed with pandas (v2.3.2) and NumPy (v1.26.4). Preprocessing relied on the NLTK library (v3.9.1) for tasks such as sentence segmentation. For sentence embeddings, we used the Qwen/Qwen3-Embedding-0.6B model via the SentenceTransformers library (v5.1.0). The core TM was conducted using BERTopic (v0.17.3), which leveraged UMAP (umap-learn v0.5.9.post2) for dimensionality reduction and HDBSCAN (hdbscan v0.8.40) for clustering. We used the Optuna framework (v4.5.0) to perform a Bayesian optimization of hyperparameters. Topic labelling was automated using a local instance of the Llama 3 model, accessed via the Llama-CPP-Python (v0.3.16) and Hugging Face Hub (v0.34.4) libraries. Visualizations of the topics and embeddings were created using BERTopic’s native Plotly-based functions (v6.3.0) and the datamapplot library (v0.6.4).

## Results

### High Sensory dataset: topic modelling and representation

**Fig. 2.**
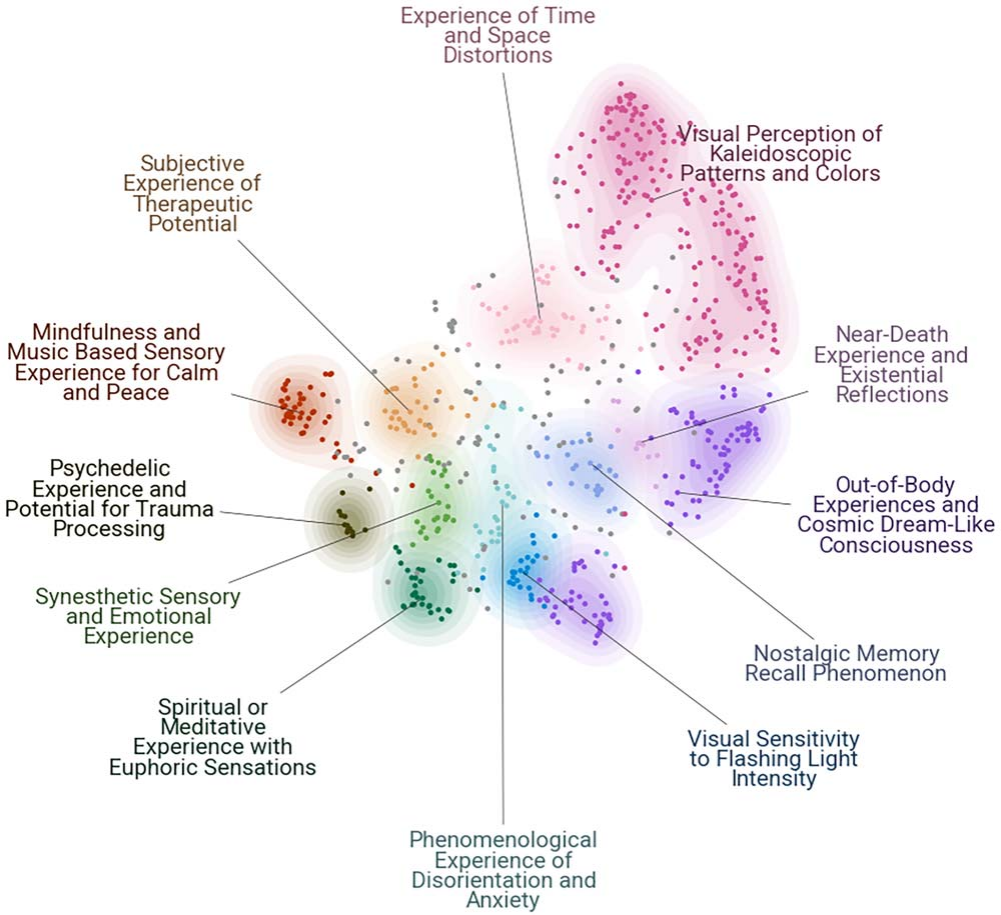
**Topic representations of HS Dreamachine experiences**: two-dimensional embedding visualization of experiential topics ($n=12$) derived from HS Dreamachine participant reflections ($n=700$ sentences), where each point represents a sentence, colour-coded by its dominant topic, spatial proximity indicates semantic similarity between reports, computed using BERTopic’s transformer-based embeddings, topic labels were generated using Llama-3-8b-instructto interpreted the underlying semantic clusters and overlapping regions suggesting shared phenomenological features.

**Fig. 3.**
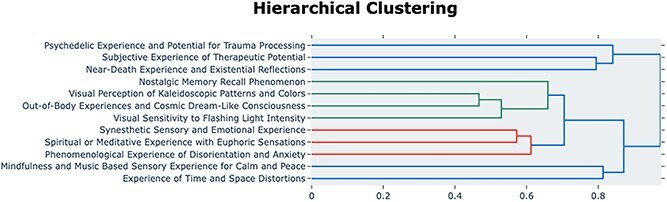
**Hierarchical clustering of participant’s reflections of the High Sensory Dreamachine condition** Dendrogram showing the hierarchical clustering of semantic relationships between the 12 topics identified from the High Sensory reports, performed on the topic centroids in the 20D UMAP space with vertical distances representing Cosine dissimilarity, revealing phenomenologically coherent domains including transformative states (e.g. Psychedelic Experience, Near-Death Experience), complex perceptual and mnemonic experiences (e.g. Visual Perception, Nostalgic Memory), and altered sensory and self-perceptions (e.g. Synesthetic Experience, Disorientation and Anxiety), complementing the spatial relationships shown in Fig. 2.

TM of participants’ High Sensory Dreamachine reflections revealed 12 distinct experiential topics, after accounting for outliers (which in BERTopic are designated as ‘topic -1’ and represent sentences that could not be confidently assigned to any cluster). These topics were automatically labelled by Llama 3 based on sets of keywords extracted from c-TF-IDF, KeyBERT representations, and MMR and representative sentences assigned for each cluster, ensuring minimal researcher bias in the labelling. Figures 2 and 3 display the relationships among these topics through 2D embedding visualization and hierarchical clustering, respectively. Spatial proximity in Fig. 2 indicates thematic similarity, and the dendrogram (Fig. 3) quantifies relationships through cosine distances (0-1, lower values indicating stronger connections).

In the spatial embedding (Fig. 2), the proximity between points suggests semantic relationships between these experiential aspects, with some overlap between domains potentially indicating shared phenomenological features.

Analysis revealed 12 (automatically labelled) distinct experiential topics:

1. **Visual Perception of Kaleidoscopic Patterns and Colours** ($n=191$ sentences): The largest topic, capturing detailed reports of geometric and colourful visual patterns, such as, *‘intense geometric shapes spiralling into a deep ocean blue circle’* and *‘shockingly vivid white and red and black, angular kaleidoscope with 3D elements’*.
2. **Out-of-Body Experiences and Cosmic Dream-Like Consciousness** ($n=125$ sentences): Characterized by experiences of disembodiment and journeys through space, with similarities with dreams experiences, with reports like *‘I felt like I was slowly floating upwards’* and *‘Felt my body was being lifted and travelling through the universe’*, *‘Dreaming while awake—flashes of random places I have been’*.
3. **Experience of Time and Space Distortions** ($n=58$ sentences): Describes alterations in the subjective flow of time or the perception of spatial dimensions, e.g. *‘A sense of moving through a portal, being transported’*, *‘No sense of time’* and *‘A time and a place that seem to have happened but has yet to come’*.
4. **Mindfulness and Music Based Sensory Experience for Calm and Peace** ($n=54$ sentences): Captures feelings of deep relaxation and tranquillity, often linked to the music, as in *‘Connection to an inner peace’*, *‘A feeling of peace and tranquillity’*, *‘The music is calming and surreal’*.
5. **Spiritual or Meditative Experience with Euphoric Sensations** ($n=40$ sentences): Contains reports of profound spiritual or blissful states, such as *‘I felt an overwhelming sense of euphoria and love for everyone in the room’*, *‘I felt very peaceful, euphoric, with a sense of wonder and awe’*.
6. **Subjective Experience of Therapeutic Potential** ($n=33$ sentences): Characterized by reflections on the healing aspects of the experience, for instance, *‘It felt very healing and a very emotional experience’*, *‘Very ecstatic experience that I wished would never end’*.
7. **Nostalgic Memory Recall Phenomenon** ($n=29$ sentences): Describes the emergence of vivid, autobiographical memories, such as *‘The most vivid memories of my Dad carrying me on his shoulders’*, or *‘I went back to many hard and mostly beautiful memories without prompting them at all’*.
8. **Synesthetic Sensory and Emotional Experience** ($n=27$ sentences): Captures the blending of senses, e.g. *‘Sound created the visuals and created a pattern of energy movement across my body’*, *‘And this made me think about synesthetic experiences and how they’re very precious and worth seeking out and appreciating’*.
9. **Phenomenological Experience of Disorientation and Anxiety** (n=24 sentences): Includes reports of confusion or challenging sensory experiences, such as *‘I felt intensely anxious and a bit suffocated’*, *‘Anxious and overwhelmed, total lack of control at first’*.
10. **Visual Sensitivity to Flashing Light Intensity** (n=24 sentences): Contains reports focused on the direct physiological reaction to the light itself, e.g. *‘Bright light was a bit hurting’*, *‘I saw the inside of my eyelids in a whole new way’*.
11. **Psychedelic Experience and Potential for Trauma Processing** (n=14 sentences): Characterized by explicit comparisons with psychedelic drug experiences, as in *‘this is exactly how I remember being on LSD was, without the scary parts’*, or *‘The closest thing I’ll ever experience to being on drugs’*.
12. **Near-Death Experience and Existential Reflections** (n=13 sentences): Describes profound experiences with existential themes, such as *‘It kind of felt like dying, reincarnating, and starting life again’* or *‘What I imagine it’s like looking back on life before you die’*.

The hierarchical clustering analysis (Fig. 3) organizes the 12 experiential topics into distinct phenomenological groupings based on their cosine dissimilarity. The analysis reveals a higher-order cluster of transformative states, where *‘Psychedelic Experience and Trauma Processing’* and ‘*Subjective Experience of Therapeutic Potential*’ are linked first. This pair is subsequently joined by ‘*Near Death Experience and Existential Reflections*’. A second large branch groups several topics, including a pairing of *‘Spiritual or Meditative Experience with Euphoric Sensations’* and *‘Phenomenological Experience of Disorientation and Anxiety’*. Another notable sub-cluster in this branch links *‘Visual Perception of Kaleidoscopic Patterns and Colours’* with *‘Out-of-Body Experiences’ and ‘Nostalgic Memory Recall Phenomenon’*. A final, distinct pairing connects *‘Mindfulness and Music Based Sensory Experience for Calm and Peace’* with *‘Experience of Time and Space Distortions’*.

The distribution of sentences across topics shows considerable variation (Fig. 2, Table 4). The dominant topics (>100 sentences) were *Visual Perception of Kaleidoscopic Patterns and Colours* ($n=191$ sentences) and *Out-of-Body Experiences and Cosmic Dream-Like Consciousness* ($n=125$ sentences), suggesting these were core elements of the Dreamachine experience. A broad range of moderate-frequency topics (25–80 sentences) was also identified, including *Experience of Time and Space Distortions* ($n=58$), *Mindfulness and Music Based Sensory Experience for Calm and Peace* ($n=54$), *Spiritual or Meditative Experience with Euphoric Sensations* ($n=40$), *Subjective Experience of Therapeutic Potential* ($n=33$), *Nostalgic Memory Recall Phenomenon* ($n=29$), and *Synesthetic Sensory and Emotional Experience* ($n=27$). Finally, lower-frequency topics (<25 sentences) consisted of *Phenomenological Experience of Disorientation and Anxiety* (n=24), *Visual Sensitivity to Flashing Light Intensity* (n=24), *Psychedelic Experience and Potential for Trauma Processing (n=14)*, and *Near-Death Experience and Existential Reflections* (n=13).

**Table 4:** Distribution of experiential topics in High Sensory Dreamachine open reports

**Topic label**	**Sentence count**	**Percentage (%)**
Visual perception of kaleidoscopic patterns and colours	191	27.29
Out-of-body experiences and cosmic dream-like consciousness	125	17.86
Outliers	68	9.71
Experience of time and space distortions	58	8.29
Mindfulness and music based sensory experience for calm and peace	54	7.71
Spiritual or meditative experience with euphoric sensations	40	5.71
Subjective experience of therapeutic potential	33	4.71
Nostalgic memory recall phenomenon	29	4.14
Synesthetic sensory and emotional experience	27	3.86
Phenomenological experience of disorientation and anxiety	24	3.43
Visual sensitivity to flashing light intensity	24	3.43
Psychedelic experience and potential for trauma processing	14	2.00
Near-death experience and existential reflections	13	1.86
Total	700	100.00

### Deep Listening dataset: topic modelling and representation

**Fig. 4.**
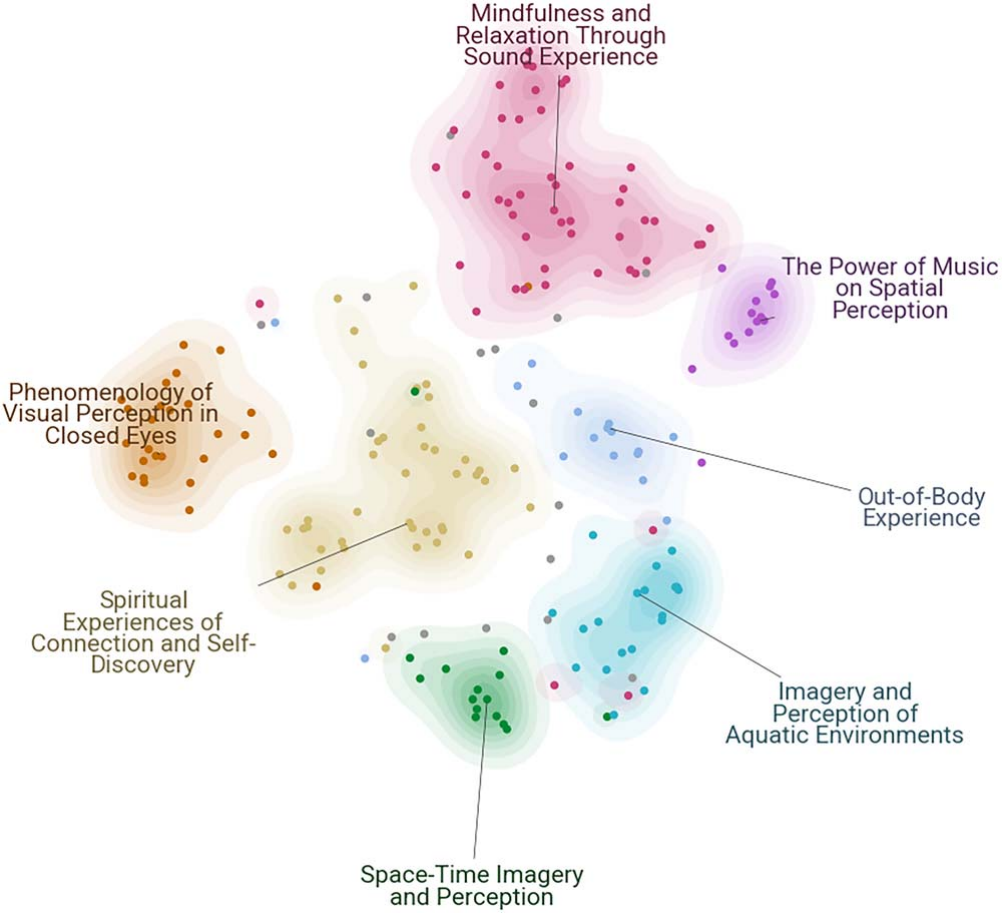
**Topic representations of Deep Listening Dreamachine experiences** Two-dimensional embedding visualization of experiential topics (n=7) derived from Deep Listening Dreamachine participant reflections (n=198 sentences), where each point represents a sentence, colour-coded by its dominant topic, with spatial proximity indicates semantic similarity between reports, computed using BERTopic’s transformer-based embeddings.

The TM analysis of participants’ reflections following the Deep Listening Dreamachine yielded seven distinct topics (Fig. 4, Table 5). These topics, automatically identified by BERTopic and labelled by Llama 3, capture a range of sensory, cognitive, and emotional dimensions, and are detailed below with their corresponding sentence counts and example of representative quotes:

1. **Mindfulness and Relaxation Through Sound Experience** (n=50 sentences): The most dominant topic, focusing on deep calm and meditative states, with reports like *‘Complete relaxation, peace of mind and ready to face whatever happens’*, *‘Relaxed and peaceful; felt the beats of the music’*.
2. **Spiritual Experiences of Connection and Self-Discovery** (derived from n=41 sentences): Captures introspective journeys and feelings of insight, e.g. ‘*I saw past experiences and reflected on my choices*’, *‘I began to see the source, the light we all come from, the connection to everything’*.
3. **Phenomenology of Visual Perception in Closed Eyes** (n=27 sentences): Describes the minimal visual phenomena experienced, such as ‘*The colours behind my closed eyes swapped to the opposite colour of the colour spectrum*’, *‘The colours made patterns when I had my eyes closed—almost like a weave of colours in a woollen fabric’*.
4. **Imagery and Perception of Aquatic Environments** (n=19 sentences): Imagery-based topic involving water or oceanic scenes, with reports like *‘It felt like I was deep in the ocean’*, *‘I turned into a mermaid and watched them on the land as I swam away with turtles and fishes’*.
5. **Out-of-Body Experience** (n=16 sentences): Contains reports of disembodiment or floating, for instance,*‘I felt like my body kept sinking or dropping to another level... into the seat and into itself’*, *‘Detachment, as though my body was in one box, mind in another’*.
6. **Space-Time Imagery and Perception** (n=15 sentences): Characterized by reports of dream-like journeys, such as *‘I saw everything, the universe, the multiverse, all of time’*, *‘Flying through a lot of shapes, light, space’*.
7. **The Power of Music on Spatial Perception** (n=14 sentences): Focuses on how the music influenced the sense of space and immersion, captured in reports like *‘Music was not just in the space, it was the space’*, *‘I watched the room rise and fall with the music’*.

**Table 5:** Distribution of experiential topics in Deep Listening (DL) Dreamachine reports

**Topic label**	**Sentence count**	**Percentage (%)**
Mindfulness and relaxation through sound experience	50	25.25
Spiritual experiences of connection and self-discovery	41	20.71
Phenomenology of visual perception in closed eyes	27	13.64
Imagery and perception of aquatic environments	19	9.60
Out-of-body experience	16	8.08
Outliers	16	8.08
Space-time imagery and perception	15	7.58
The power of music on spatial perception	14	7.07
Total	198	100.00

**Fig. 5.**
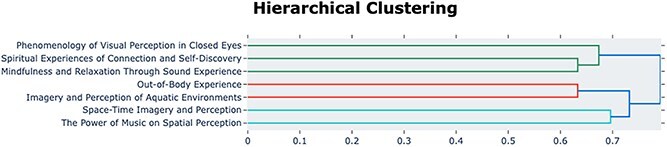
**Hierarchical clustering of DL Dreamachine experiences** Dendrogram showing semantic relationships between seven topics identified from the Deep Listening version.

The hierarchical clustering analysis (Fig. 5) provides a more quantitative view of the relationships between these topics. The analysis reveals two primary branches. One branch groups together the three topics related to imagery and altered self-perception: ‘*Out-of-Body Experience*’ pairing with ‘*Imagery and Perception of Aquatic Environments*’, which is subsequently joined by ‘*Space-Time Imagery and Perception*’. The other, larger branch contains the remaining topics, with ‘*Mindfulness and Relaxation*’ and ‘*Spiritual Experiences*’ showing a closer link.

A preliminary comparison between High Sensory and Deep Listening (Figs 4 and 5) versions reveals both shared themes and unique characteristics. For instance, both conditions produced topics related to out-of-body or ‘cosmic-like’ journey experiences. However, the High Sensory condition is uniquely characterized by intense topics such as ‘Psychedelic Experience’ and ‘Near-Death Experience’, which have no direct equivalent here. Conversely, the Deep Listening condition yielded a topic focused specifically on the auditory experience, *The Power of Music on Spatial Perception*. It is important to note that differences in topic labels may reflect genuine variations in the nature of participants experiences across these two versions of the Dreamachine (though some minor differences may result from the stochastic, generative nature of Llama 3). Conversely, similar topic labels do not necessarily indicate similar experiences, as participants may use overlapping language to describe qualitatively distinct states. For example, a ‘dream-like state’ could emerge both from listening to music with eyes closed and from the combination of music and SLS, yet these experiences may differ significantly in their underlying phenomenology.

### Quantitative comparison of experiential themes across conditions

To quantify the observed differences between the High Sensory and Deep Listening conditions, we compared topics based on their semantic contents (embedding similarity) rather than their label similarity. Direct label matching is unreliable because topic solutions can differ across runs and corpora (algorithmic stochasticity, dataset size/vocabulary), and because hyperparameters were optimized separately per condition. We addressed this by first computing a ‘semantic centroid’ for each topic by averaging the sentence embeddings of its constituent sentences, using the same Qwen/Qwen3-Embedding-0.6B model for consistency. We then measured cross-condition similarity between the centroids. This technique is well supported as a robust measure of semantic proximity in high-dimensional contextual space (Angelov 2020, Reimers & Gurevych 2019, Grootendorst 2022). The resulting HS$\times $DL similarity matrix revealed five high-similarity cross-condition pairs above a prespecified threshold of $\tau =0.85$.

Based on the resulting similarity matrix (see Figure S3) we constructed 1:1 greedy matches by iteratively selecting the highest-similarity High Sensory-Deep Listening pair exceeding the threshold $\tau =0.85$ and removing both topics before the next iteration. This yielded five paired ‘experiential themes’: (i) ‘Mindfulness/music-based calm’ with ‘Mindfulness/relaxation through sound’ (0.96), (ii) ‘Out-of-body/cosmic experience’ with ‘Out-of-body experience’ (0.94), (iii) ‘Nostalgic memory’ with ‘Spiritual connection/self-discovery’ (0.917), (iv) ‘Visual sensitivity to flashing light’ with ‘Closed-eye visual phenomenology’ (0.911), and (v) ‘Time and space distortion’ with ‘Space-time imagery’ (0.869), plus seven High Sensory-specific themes and two Deep Listening-specific themes. These pairs and unmatched topics were combined into a merged contingency table for inference, as detailed in Table S1.

**Fig. 6.**
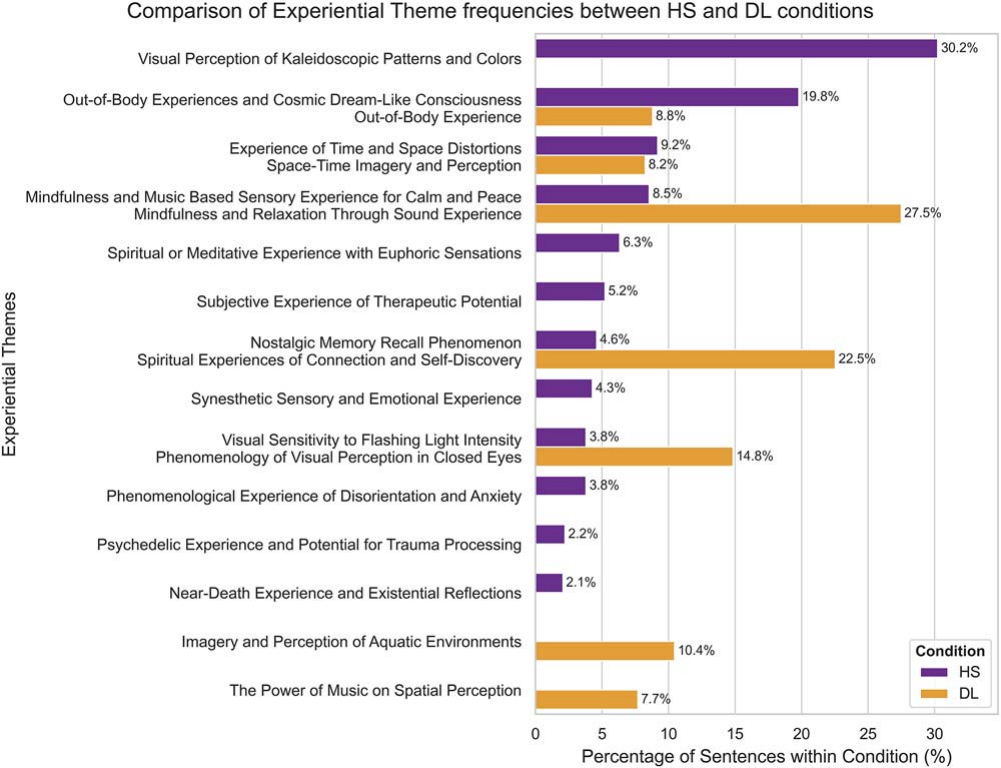
**Comparative bar chart of experiential theme frequencies between the High Sensory and Deep Listening conditions.** Bar chart displaying the frequency of experiential themes, normalized as a percentage of the total sentences within each condition, where the themes on the y-axis are a merged set, derived by calculating the cosine similarity between the average sentence embeddings of all topics from both conditions -- with topic pairs with a semantic similarity score greater than 0.85 were considered ‘paired’ and combined into a single theme and unmatched themes shown as unique to their original condition -- and a Chi-Squared test performed on the raw counts (contengency table) confirmed a statistically significant association ($P <.001$) between the condition and the distribution of reported themes.

A Chi-Squared test of independence performed on the contingency table of the merged-themes sentence counts indicated a highly significant association between condition and theme prevalence $\chi ^{2}(13)=343.05,P<.001$. Figure 6 visualizes these differences as percentages of experiential theme prevalence within each condition. While this analysis highlights a significant difference in prevalence, these results should be interpreted cautiously given the imbalance in sample size between the two conditions.

## Discussion

To advance our understanding of stroboscopically induced phenomenology, beyond traditional categorizations, we employed a data-driven approach combining TM and LLM to analyse participants’ unstructured free text reports from the Dreamachine multisensory immersive experience. This approach addressed the limitations of structured questionnaires, which may constrain or bias the capture of diverse phenomenological experiences. The principal contribution of this work is the adaptation of TM and LLM-based approaches for phenomenological data and context, specifically, how to optimise TM parameters for phenomenological data and how to adapt the pipeline for comparing experiential reports across conditions. The empirical application to the Dreamachine dataset therefore serves a dual purpose, validating the pipeline’s interpretive power and illustrating how this computational approach to phenomenology can help to systematically reveal structured experiential dimensions within complex, large-scale, open-ended reports.

### Phenomenology of the Dreamachine

Our analysis of free-text reports from the Dreamachine revealed a spectrum of stroboscopically induced phenomenology broader than that documented in previous laboratory studies. Our findings identified a rich variety of phenomenological reports, which for clarity of discussion, we have organized into three principal domains suggested by our data: simple visual hallucinations, which are consistent with the existing literature; complex (often visual) experiences involving semantic content and narrative features; and ASCs. In the following sections, we will discuss each of these categories, carefully distinguishing between the findings that are directly grounded in our topic analysis and the more speculative, theory-driven interpretations that can help contextualize these results.

#### Simple visual hallucinations

Simple visual hallucinations encompass a range of basic visual hallucinations characterized by their lack of narrative content. These phenomena include phosphenes, geometric forms, vivid colours, and fractal or kaleidoscopic patterns (Smythies 1959, Shevelev et al. 2000, Bressloff et al. 2002, Rule et al. 2011). Their classification as ‘simple’ stems from their abstract nature rather than visual complexity (e.g. fractals).

The prevalence of simple visual hallucinations in the High Sensory version of Dreamachine is consistent with the established literature on SLS-induced phenomena (Young et al. 1975, Allefeld et al. 2011, Billock and Tsou 2012). This was directly supported by our results, where the largest identified topic was ‘*Visual Perception of Kaleidoscopic Patterns and Colours*’ (n=191 sentences), capturing reports of vibrant geometric patterns, kaleidoscopic visuals, and dynamic colour phenomena. Example reports like ‘*shockingly vivid white and red and black, angular kaleidoscope with 3D elements*’ are good illustrators of this category.

These experiences likely originate from altered perceptual processing in lower-level visual areas, particularly the primary visual cortex (V1) (Bressloff et al. 2002, Kometer and Vollenweider 2018). Neural field models posit that simple visual hallucinations should be organized around the centre of the visual field, where the highest level of detail occurs, as exemplified by the tunnel, spiral, and target Klüver forms (Bressloff et al. 2002). These models align with the architecture and function of V1, where neurons are organized into hypercolumns responding to specific orientations and spatial frequencies (Rule et al. 2011). The architecture of V1 is therefore believed to be a good candidate underlying the perception of the specific reported geometric properties of simple visual hallucinations, such as grids, lattices, and spirals. SLS may lead groups of neurons in V1 to fire in synchrony, inducing resonance in these neural populations, leading to the perception of geometric hallucinations (Billock and Tsou 2012). This resonance effect could explain the prevalence of certain geometric forms in stroboscopically induced simple visual hallucinations, with the most commonly reported forms likely corresponding to the most stable or easily induced patterns of neural activity in V1.

#### Complex visual hallucinations

Beyond simple patterns, our analysis revealed the frequent reporting of complex visual experiences, which we define here as those containing meaningful, recognizable, or narrative content. This is directly evidenced by the emergence of several distinct topics, such as ‘Nostalgic Memory Recall Phenomenon’ (e.g. ‘The most vivid memories of my Dad carrying me on his shoulders’), ‘Out-of-Body Experiences and Cosmic Dream-Like Consciousness’ (e.g. ‘Dreaming while awake-flashes of random places I have been’), and ‘Near-Death Experience and Existential Reflections’ (e.g. ‘What I imagine it is like looking back on life before you die’). The content of these topics, grounded in participants’ own words, strongly indicates that the experience involved higher-level cognitive processing, incorporating elements from memory, imagination, and personal narrative. As a note of caution, we acknowledge the important, albeit often blurry, distinction between such experiences and vivid, spontaneous mental imagery. While hallucinations are sometimes strictly defined by a sense of being externally real, the term is also widely used to describe internally generated, eyes-closed experiences that possess a strong perceptual quality, akin to ‘seeing’.

Having established the presence of complex experiences in our data, we now turn to more speculative interpretations of possible neural and cognitive mechanisms that may underlie them. Several factors may contribute to the generation of complex visual experiences during SLS. From a neural perspective, SLS may disrupt the balance of excitatory and inhibitory activity in early visual cortices, subsequently triggering downstream activation of higher-order visual areas including the ventral temporal cortex and associative regions. This pattern of hierarchical activation could explain the progression from simple geometric patterns to more complex, meaningful percepts. Such distributed activation is likely to engage regions supporting memory retrieval (hippocampus), emotional processing (amygdala), and cognitive control (prefrontal cortex). The interaction between these neural systems may explain why complex visual hallucinations often incorporate personally meaningful content and emotionally coloured imagery.

Another possibility is that some participants who have reported complex visual experiences may have entered a drowsy hypnagogic state, which is commonly associated with highly vivid visual experiences that often lack a narrative or overarching themes (Wackermann et al. 2008, Ghibellini and Meier 2023). Given that participants were reclining, in a relaxed setting, with eyes closed, and that SLS have been previously associated with vigilance reduction (Bartossek et al. 2021), it is possible that some of the reported complex visual experiences could be related to hypnagogia.

Moreover, it is possible that these neural and cognitive processes may be further modulated by top-down influences, particularly through predictive processing mechanisms where prior expectations shape perceptual inference (Powers et al. 2016).

Beyond these potential neural and cognitive processes at play, interindividual differences may further modulate the likelihood and character of complex visual hallucinations. One relevant candidate factor is visual mental imagery, which is defined as the cognitive capacity to reactivate and manipulate visual representations in the absence of corresponding external visual input (‘seeing with the mind’s eye’) (Ganis and Schendan 2011, Dijkstra et al. 2017). Individuals with stronger visual imagery may be more likely to report complex visual hallucinations, as their internally generated imagery could be incorporated into the stroboscopic experience. Indeed, in healthy individuals, heightened mental imagery has been shown to increase the probability of experiencing mild visual or auditory hallucinations (Barrett and Etheridge 1992, Aleman et al. 2000). This connection is also suggested in clinical contexts, where stronger imagery is associated with more frequent hallucinations in patients with schizophrenia (Mintz and Alpert 1972, Matthews et al. 2014) and Parkinson’s disease (Shine et al. 2015). Conversely, studies using Ganzflicker stimulation found that individuals with weaker mental imagery reported less vivid and less complex hallucinations (Königsmark et al. 2021, Reeder 2022).

Other psychological traits related to hypnotic suggestibility may also play a part, such as absorption, which relates to the tendency to become immersed in experiences (Bartossek et al. 2021), and phenomenological control (Lush et al. 2021), which refers to an individual’s ability to alter what they experience, both within and outside of the hypnotic context, in ways that are consistent with their intentions and goals. These traits may exert their effects through participants’ implicit or explicit expectations (demand characteristics) about the Dreamachine experience. Trait phenomenological control scores are known to predict subjective responses in various experimental contexts where demand characteristics are evident, such as the rubber hand illusion and Mirror-touch synaesthesia (Lush et al. 2020). Given that most participants were aware they might experience visual hallucinations, these expectations could have introduced demand characteristics, thereby influencing how they experienced and reported their experiences and survey responses, in ways consistent with the effects of phenomenological control. Future studies should test this hypothesis by screening participants, e.g. using the Phenomenological Control Scale (Lush et al. 2021), to examine individual differences in phenomenological control and their potential predictive role in the occurrence of both complex and simple visual hallucinations, and other commonly reported phenomena in strobe-induced altered states.

#### Altered states of consciousness

ASC experiences describe mental states that differ profoundly from typical, everyday states of consciousness. These experiences may include alterations in how the external world is perceived, variations in the awareness of one’s emotions, sensations, and thoughts, a distorted sense of space and time, changes in self-awareness including ego dissolution, or feelings of unity or oneness with the environment (Dittrich 1998, Studerus et al. 2010, Schmidt and Berkemeyer 2018).

A key finding of our study, emerging directly from the data-driven analysis, was the presence of multiple topics explicitly describing ASCs. This was particularly evident in the High Sensory dataset, which included topics such as ‘Psychedelic Experience and Potential for Trauma Processing’ (e.g. ‘this is exactly how I remember being on LSD was’), ‘Out-of-Body Experiences and Cosmic Dream-Like Consciousness’, ‘Experience of Time and Space Distortions’, and ‘Spiritual or Meditative Experience with Euphoric Sensations’. The emergence and content of these distinct topics provide strong, bottom-up evidence that a significant portion of participants reported experiences that go far beyond simple visual phenomena. To our knowledge, ASC reports like these have not typically been observed in laboratory SLS studies (Bartossek et al. 2021).

While potentially sharing some neural mechanisms with complex visual hallucinations, ASCs may represent a broader alteration of consciousness that extends beyond visual processing. The naturalistic setting of the Dreamachine, compared with laboratory SLS studies, may create different expectational contexts that influence both complex visual hallucinations and ASCs, though potentially in distinct ways. For instance, while complex visual hallucination expectations might focus on specific visual content, ASC expectations may relate more to overall state changes. These different types of expectations could engage distinct neural and cognitive mechanisms, despite potentially overlapping neural substrates (Howard et al. 1998, Wittmann et al. 2007, Ffytche 2008, Kometer et al. 2013). The interaction between bottom-up and top-down processes in generating these experiences may depend on various factors, including expectations, naturalistic set-up, and neural entrainment (Carhart-Harris et al. 2018b, Carhart-Harris and Friston 2019, Schwartzman et al. 2019, Amaya et al. 2023). Our study’s contribution is to empirically demonstrate that under certain conditions, the phenomenology of SLS can extend beyond visual effects to encompass profound alterations of consciousness, opening new avenues for investigating how contextual factors modulate the effects of sensory stimulation. Future studies should systematically investigate how these contextual factors and individual differences influence the emergence of both complex visual hallucinations and ASCs during SLS.

Moreover, although not explicitly identified as distinct topics, an examination of individual sentence structures revealed that many reports conveyed emotional valence. Participants also described cognitive changes, including elements of self-reflection and autobiographical memory. Additionally, and likely due to the 360-deg music participants were listening to, auditory experiences were frequently emphasized as an important part of the experience, aligning with the established additive effects of music and stroboscopic stimulation (Montgomery et al. 2024). Interestingly, these aspects of experience bear some resemblance to findings from a previous study that applied quantitative textual analysis to reports from first-time ayahuasca experiences in ceremonies accompanied by music (Cruz et al. 2023).

### Methodological innovation and implications

Traditional studies of ASCs and visual hallucinations have relied on structured questionnaires such as the Five-Dimensional Altered States of Consciousness Scale (5D-ASC) (Dittrich 1998, Dittrich et al. 2010, Studerus et al. 2010), the Phenomenology of Consciousness Inventory (Pekala 1991) or the Mystical Experience Questionnaire (MacLean et al. 2012). While these instruments facilitate standardized analysis, their predefined categories may overlook unexpected aspects of experience. Moreover, questionnaires can be prone to biases introduced by the framing of questions and the researchers’ preconceptions and beliefs about the phenomena being studied. In contrast, open-ended reports allow participants to describe their experiences in their own words, preserving the richness and nuance of their subjective experiences, potentially revealing novel insights. However, analysing such open-ended reports presents significant challenges. The unstructured nature of free-text responses complicates systematic analysis, and interpretation remains susceptible to researcher bias. Practically, processing and analysing open-ended reports is significantly more time-intensive compared with structured methods.

To address these challenges while retaining the benefits of open report, we used NLP models, to bridge the gap between the richness of qualitative data and the analytical power of quantitative methods. By avoiding predefined categories, our approach enables the discovery of a broader range of experiences, including those not typically associated with SLS in prior research. The data-driven nature of our approach reduces the influence of researcher preconceptions, allowing patterns to emerge from the data itself. Importantly, our approach is intended to complement rather than replace traditional methods, providing a data-driven pathway to systematically analyse phenomenological dimensions of subjective experience.

To promote reproducibility and facilitate wider adoption of this approach, we provide an open-access detailed documentation of our MOSAIC pipeline (https://github.com/romybeaute/MOSAIC), including preprocessing steps, parameter settings, and interpretation guidelines (see section 4.4). Additionally, we provide an interactive web application (https://huggingface.co/spaces/romybeaute/MOSAICapp) that enables researchers to apply the complete MOSAIC pipeline to their own datasets without requiring programming expertise, further lowering technical barriers to adoption.

### Limitations

The present study has several important limitations. First, Dreamachine’s name and marketing likely influenced participants’ expectations, which may have shaped their experiences and subsequent reports. While both High Sensory and Deep Listening versions shared contextual elements (e.g. setting, music), differences in sensory stimulation likely affected participants differently: the High Sensory condition’s stroboscopic effects may have maintained arousal, whereas the Deep Listening condition’s encouraged a deeply relaxing and potentially hypnagogic state. Moreover, the immersive soundscape in both versions, the guided pre-experience meditation, the postexperience time for reflection, and the fact that participants undertook the experience simultaneously in groups of 20–30 people likely contributed significantly to the emotional depth and cognitive engagement reported in their individual experiences, and could also have contributed to similarity/convergence among reports. While such shared context may not entail a ‘collective experience’ in the stricter sense, it has the potential to influence individual phenomenology, as demonstrated by research on collective rituals and shared experiences showing that such settings enhance emotional synchrony and feelings of connectedness among participants (Páez et al. 2015, Carhart-Harris et al. 2018a, Kettner et al. 2021). Beyond the potential experiential influences of the social setting, the group context may also have shaped how experiences were interpreted and described, as participants could discuss the experience with one another immediately afterwards. Such post-experience interaction could therefore have reduced interindividual variability in reports, amplifying shared themes that might not have emerged as strongly in solitary settings. It is therefore important to recognize that while SLS may enhance or catalyse different types of stroboscopically induced phenomenology, other factors such as set, setting, and individual differences likely play crucial roles in shaping reports of ASC and complex visual hallucinations (Carhart-Harris et al. 2018b).

A second limitation of this study concerns the sample size of Dreamachine’s open reports dataset, relative to the total visitor population ($\sim $40 000). This disparity likely results from the data collection methodology, wherein phenomenological reports were collected through an optional free-text question positioned at the end of a comprehensive digital survey that was one of a number of reflection options available to participants, and limited by availability. Prior to this final open reflection question (‘In the Dreamachine I experienced...’), participants had already responded to targeted open-ended questions focusing on specific aspects of their experience, including emotions, visual phenomena, auditory experiences, cognitive changes, and bodily sensations. Consequently, participants may have felt they had already thoroughly documented their experience and opted not to provide additional commentary in the final open reflection, which was the focus of our present analysis. To minimize prompt-related biases, and capture a more global account of the entire experience, we analysed only the final open-ended reflections, rather than aggregating multiple free-text responses that could have skewed the results. This approach aimed to capture spontaneous reporting of the most salient aspects of participants’ experiences without being guided and influenced by specific prompts about particular phenomena. However, it also resulted in a smaller sample size, as only those particularly motivated to share in-depth reflections contributed. This self-selection may have introduced a sampling bias, as participants who provided detailed written responses may differ systematically from those who did not. This limitation should be considered when interpreting the generalizability of our findings to the broader Dreamachine population.

A third limitation relates to the data collection interface itself. As noted in the methods, the open-ended reflection analysed here (‘In Dreamachine I experienced...”) was presented to participants after they had already answered more targeted questions about specific sensory and emotional aspects of their experience. This structure may have created a priming effect, encouraging participants to adopt a more metacognitive or interpretive stance in their final, general reflection. Consequently, it is difficult to fully disentangle which aspects of the reports (e.g. autobiographical memories) were spontaneously triggered by the stimulation itself versus those that arose from the process of guided reflection inherent in the survey. However, as our goal was to analyse the full phenomenological report in its final, integrated form, we consider this richness, which includes both direct perceptual content and subsequent interpretation, to be a key feature of the dataset suited to our analytical approach.

A further limitation is the absence of detailed participant demographic data, such as age or gender. This information was not collected due to the public-facing, large-scale nature of the Dreamachine exhibition, where the priority was to ensure accessibility and minimize barriers to participation, including data privacy concerns. While this prevents a more granular analysis of how experiences might vary across different demographic groups, it was a necessary trade-off for the project’s design.

From a methodological perspective, the TM process has inherent limitations regarding stability and parameter selection. To mitigate the subjectivity of parameter choice, we employed a Bayesian optimization procedure with the Optuna framework, systematically tuning hyperparameters across 100 trials. Furthermore, to ensure that our results were not artefacts driven by algorithmic stochasticity, we conducted a bootstrapping analysis, which confirmed that semantically equivalent topics consistently re-emerge when training on random 80% subsamples of the data.

However, the selection of evaluation metrics (e.g. $C_{v}$, $C_{embed}$) to optimize for remains a decision guided by research goals, and therefore inherently includes a subjective component. Similarly, text preprocessing choices, such as sentence-level segmentation, were made to enable a detailed analysis but may have occasionally disrupted contextual coherence across sentences.

Furthermore, a key limitation lies in the inherent assumptions of the NLP models themselves. Both the sentence-transformer model and the LLM were pre-trained on massive, general-domain text datasets, not on domain-specific phenomenological reports. This means the models’ interpretation is shaped by common linguistic patterns rather than the nuanced language of subjective experience. Consequently, when faced with novel or rare descriptions such as of stroboscopic phenomena, the models likely map this language onto the closest, most familiar experiential templates from their training data (e.g. dream reports). This reliance on generalized data is a potential source of bias that should be considered when interpreting the results.

It is also important to clarify the relationship between the 2D topic visualization (Figs 2 and 4) and the hierarchical clustering (Figs 3 and 5), as they offer complementary views of the data. The 2D UMAP plot is a projection optimized to preserve the local structure between individual sentences. The hierarchical dendrogram is calculated from the global distances between the aggregated centroids of each topic cluster in the higher-dimensional space used for analysis. This global calculation is less sensitive to the nuanced, overlapping relationships visible at the sentence level in the main Figs 2 and 4. This methodological distinction can explain why some groupings in the dendrogram may appear counter-intuitive when compared with the 2D plot, as each visualization emphasizes a different aspect of the topics’ underlying semantic structure.

Additionally, it is important to address the interpretation of the automatically generated topic labels. Terms such as ‘dream state” or ‘lucid dreaming state” were produced by the LLM and retained to preserve the objectivity of our data-driven approach and avoid introducing researcher bias through manual relabelling. However, these labels should not be interpreted as ground-truth classifications of physiological states. Instead, they should be understood as indicators of semantic similarity; given that reports of stroboscopic stimulation are rare, the model likely maps these novel experiences onto more common phenomenological templates from its training data, such as dreams. The labels are therefore useful pointers to the phenomenological character of the reports, rather than definitive statements about the underlying state. To generate labels that are as contextually-grounded as possible, we enhanced our pipeline to use multiple keyword extraction techniques, as detailed in the methods.

Finally, it is critical to distinguish between semantic domains and propositional assertions in TM. Algorithms cluster sentences based on semantic proximity in vector space. Consequently, a report stating ‘I did not see patterns’ may cluster with ‘Visual Patterns’ because the subject matter is identical, despite the negation. Similarly, metaphorical descriptions (e.g. ‘It was like dying’) cluster with literal descriptions of the same theme. However, the phrasing of the reflection prompt (‘In Dreamachine I experienced...’) was designed to elicit affirmative experiential reports, making negations and non-experiential content unlikely in practice. Nonetheless, topics should be interpreted as reflecting the presence of discourse concerning these themes, rather than as evidence that a specific experiential state (e.g. a Near-Death Experience) was achieved.

### Future directions

Our study opens up several promising avenues for future research. While our current analysis reveals distinct aspects of experiences during SLS, ranging from simple geometric patterns to more complex narratives or higher-level phenomenological states, the underlying neurocognitive mechanisms remain to be fully elucidated. A critical question in this regard is how expectations and prior knowledge shape the nature and intensity of SLS experiences. Future studies could systematically investigate this through expectation manipulation paradigms and measurement of trait suggestibility or phenomenological control, allowing researchers to determine which experiential reports are contextually influenced and which remain stable across varying expectations.

To ensure the robustness and generalizability of these findings, replication studies using varied SLS set-ups are needed. Future research should explore how different SLS frequencies, luminance, durations, and accompanying stimuli affect the types of experiences reported. Methodologically, future studies should focus on developing more robust methods for evaluating the stability and quality of NLP-derived topics in the context of subjective experience research. This could include the development of specialized coherence metrics tailored for phenomenological data. Furthermore, a next important step will be to directly compare the results from the structured questionnaires with the themes identified by our pipeline. This will allow for a future assessment of what our data-driven method reveals ‘beyond standard questionnaires’ and help validate the complementary strengths and links of both approaches. In addition to these empirical directions, our NLP approach offers a systematic categorization of stroboscopically induced phenomenology through an open-source TM pipeline. This categorization provides well-defined phenomenological targets that could be used in future neuroimaging studies of stroboscopically induced phenomenology to investigate whether different experiential categories (e.g. geometric patterns versus spiritual states) correspond to distinct patterns of brain activity or network connectivity. Establishing such mappings would help advance our understanding of the relationship between subjective experiences and their neural correlates.

More generally, this TM pipeline, which combines BERTopic for semantic clustering and Llama for topic interpretation, offers a systematic, reproducible framework for analysing free-text phenomenological data. The workflow encompasses five key stages: (i) data preprocessing and cleaning, (ii) semantic embedding generation, (iii) TM and clustering, (iv) LLM-assisted topic interpretation, and (v) hierarchical relationship analysis.

While our approach largely builds on existing text analysis frameworks, it offers a specific implementation tailored for phenomenological research. While requiring careful consideration of parameters and potential modifications for different datasets, the modular nature of our approach allows researchers to adapt each component to their specific research needs. To lower technical barriers to implementation, we provide a fully documented implementation of our analytical workflow, from preprocessing to interpretation. Beyond research on SLS, this approach demonstrates the broader potential of computational methods for analysing qualitative data in consciousness studies and other domains where systematic analysis of subjective reports is valuable.

## Conclusion

Our study makes three primary contributions. First, through NLP of Dreamachine experience reports, we identified categories of experience that traditional questionnaire-based studies of stroboscopic stimulation may have overlooked, particularly in relation to complex hallucinations and ASCs. While the specific role of SLS versus contextual influences remains to be determined, our findings reveal a richer range of phenomenological experiences than previously documented in laboratory settings. Second, we demonstrate how combining established NLP techniques can effectively bridge qualitative and quantitative approaches in consciousness research and beyond. Our implementation shows that these methods can systematically analyse rich, subjective experiences, offering a plug-and-play method for identifying neurophenomenological explanatory targets. Third, by providing a fully documented, open-source analytical pipeline alongside an interactive web application, we offer researchers concrete tools to implement similar analyses in their own investigations of phenomenological data. This practical contribution lowers the technical barriers for applying systematic text analysis methods to phenomenological data. Our detailed methodology, open-source pipeline (https://github.com/romybeaute/MOSAIC), and interactive web application (https://huggingface.co/spaces/romybeaute/MOSAICapp) provide accessible tools and guidelines for researchers to adapt and apply these techniques to diverse phenomenological datasets.

## Supplementary Material

annex_niag008
